# Editorial: Development, assessment, improvement, and standardization of methods in herbal drug research

**DOI:** 10.3389/fphar.2022.1071194

**Published:** 2022-11-16

**Authors:** Abdul Rohman, Gunawan Indrayanto, Kornkanok Ingkaninan

**Affiliations:** ^1^ Department of Pharmaceutical Chemistry, Faculty of Pharmacy and Center of Excellence, Institute for Halal Industry and Systems, Universitas Gadjah Mada, Yogyakarta, Indonesia; ^2^ Faculty of Pharmacy, Universitas Surabaya, Surabaya, Indonesia; ^3^ Centre of Excellence in Cannabis Research, Department of Pharmaceutical Chemistry and Pharmacognosy, Faculty of Pharmaceutical Sciences and Center of Excellence for Innovation in Chemistry, Naresuan University, Phitsanulok, Thailand

**Keywords:** herbal drug, chemometrics, fingerprinting profiles, metabolomics, biological activities

Herbal drugs (HDs) or herbal medicines have been applied for a very long time as natural remedies for preventing and curing diseases and for improving human health. HDs are currently gaining increasing popularity globally as drugs, complementary and alternative medicines, nutraceuticals, food supplements, and cosmetics. HDs have gained wider interest among societies during the past decades due to their broad, synergistic actions on the physiological systems and the relatively lower incidence of adverse events compared to synthetic drugs (Noviana et al.). However, HD may not be entirely safe because some studies have reported adverse effects such as nephrotoxicity, hepatotoxicity, cardiotoxicity, neurotoxicity, and skin toxicity during the administration of HDs. These adverse effects may be due to the presence of some toxic metabolites or contaminants such as aflatoxins, or due to the falsification of HDs. Therefore, the quality assurance and authenticity of HDs must be assured (Heinrich, 2015). The complexity of HDs, which typically consist of many constituents, has raised major quality issues. The bioactive compounds of extracts and/or HD preparation are typically very complex and may vary. Thus, if the exact chemical composition is not determined accurately and specifically, the reported bioactivity or pharmacological effects may not always be reproducible. Assuring high-quality HDs with reproducible quality, efficacy, and safety is a challenging task. As a consequence, the availability of appropriate analytical methods is highly required, not only for the identification and standardization of HDs but also for the detection of adulterants and contaminants (Muyumba et al., 2021). The analytical methods used for the quality control of HDs, either chemical or biological-based methods, must be official methods. Otherwise, the methods should first be validated according to the latest official guidelines. Without analytical method validation, the reliability of the data cannot be confirmed, and the results would be difficult to replicate by other scientists (Indrayanto, 2022).

The quality control and authentication of HDs are typically carried out by evaluating the biological characteristics and physicochemical properties along with microscopic and macroscopic observations. The characterization and discrimination of HDs by instrumental methods can be performed using three approaches: (1) single component analysis or targeted analysis using marker compounds responsible for biological activities, (2) fingerprint profiling (i.e., profile comparison of different HDs), and (3) metabolomic studies, either using targeted or untargeted metabolomics (Ruiz et al., 2016). Due to the large datasets obtained by these methods, the use of chemometrics is a must. Pattern recognition and multivariate calibration are among the widely used chemometrics techniques for the analysis of HDs (Zhu et al., 2014).

A total of 11 manuscripts are published in this Research Topic, including two review articles and nine original research articles. A narrative review by Noviana et al. highlights the use of metabolite fingerprint profiling based on chemical responses obtained from spectroscopy-based techniques (FT-IR and NMR spectroscopy), chromatographic techniques mainly thin layer chromatography (TLC), liquid chromatography (LC), and gas chromatography with different detection systems, electrophoretic methods, direct mass spectrometry, and DNA barcoding for the standardization and quality control of HDs. Since the instrumental analyses involve large datasets, in this review the authors also critically discuss some of the chemometrics tools applied. The combination of chemometrics of pattern recognition, multivariate calibrations, and validated instrumental methods for assisting the authentication analysis are also highlighted. 

In a systematic review presented by Ibrahim et al., *Marantodes pumilum* (Blume) Kuntze, one of the Malay traditional herbal medicines, is critically discussed. Some aspects of this herbal medicine including the identification and authentication of the plant using different methods (organoleptic, microscopic, and macroscopic evaluation along with fingerprinting profiling using FT-IR spectroscopy, LC-MS, and DNA barcoding) are thoroughly discussed. Furthermore, the phytochemical constituents of the plant with known therapeutic activity are also described.

Nine original articles explored the biological activities of HDs either as a single component or in combination. Reported investigations include *in vitro* or *in vivo* studies, identification of chemical markers in HDs, and the relationship between the biological activities of HDs and chemical markers responsible for these activities.

Hu et al. identify chemical markers in the classes of phenolics and saponins from *Eleutherococcus senticosus* (Rupr. & Maxim.) leaves and their correlation with the hypoglycemic activity is measured by α-glucosidase inhibition assay. Ultra-performance liquid chromatography combined with tandem mass spectrometry (i.e., UPLC-QTOF-MS/MS and UPLC-QTRAP-MS/MS) is validated for the qualitative and quantitative analyses of the herb. Thirty compounds are identified including seven saponins and 20 phenolic compounds. Twelve of these compounds are isolated from *E. senticosus* for the first time. The newly isolated compounds are 5-O-caffeoylshikimic acid, quinic acid butyl ester, methyl 5-O-feruloylquinate, 5-O-p-coumaroylquinic acid butyl ester, 4-O-caffeoylquinic acid methyl ester, 5-O-feruloylquinic acid, 3,4-dihydroxybenzenepropionic acid methyl ester, quercetin 3-O-β-D-glucopyranosyl-(1→6)-β-D-glucopyranoside, (7S,8R)-urolignoside, *n*-butyl-1-O-α-l-rhamnopyranoside, (Z)-hex-3-en-1-ol O-β-D-xylopyranosyl-(1″-6′)-β-D-glucopyranoside, and hexenyl-rutinoside. The phenolic compounds can be used as markers for the hypoglycemic activity of this herb.

The effect of a combination of turmeric (*Curcuma longa* L.), grape (*Vitis vinifera* L.) seed, flaxseed (*Linum usitatissimum* L.), and psyllium (*Plantago ovata* L.) with bentonite as detoxification and cholesterol-lowering agents is investigated by Turgut et al. in hypercholesterolemic mice. The combination has a synergistic effect on reducing the risk of cardiovascular diseases. The chemical markers identified in turmeric using liquid chromatography and LC-MS/MS are curcuminoids (curcumin, desmethoxycurcumin, and bisdemethoxycurcumin), while chlorogenic acid, fumaric acid, (-)-epicatechin gallate, caffeic acid, vanillic acid, luteolin 7-glucoside, resveratrol, apigenin 7-glucoside, quercetin, luteolin, and apigenin are detected in grape seed.

Furthermore, Saggam et al. study the anti-cancer activity of extracts from two Ayurveda-based immunomodulatory herbs, *Asparagus racemosus* Willd and *Withania somnifera* (L.) Dunal, for therapeutic adjuvants in countering paclitaxel (PTX)-induced myelosuppression. The studied extracts significantly modulated 20 cytokines to evade PTX-induced leukopenia, neutropenia, and morbidity. Both extracts also prevented PTX-induced myelosuppression and morbidity signs by modulating associated cytokines. The results conclude that both HDs are potential therapeutic adjuvants in cancer management.

Sun et al. investigate the effects of Shuangxinfang, a formula of Traditional Chinese Medicine (TCM), in preventing S100A9-induced macrophage/microglial inflammation in rats after acute myocardial infarction. S100A9 is an important target protein in macrophage/microglial inflammation. The authors report that Shuangxinfang could promote the recovery of cardiac function and improve depression-like behavior.

Wang et al. study the effect of Yinhuapinggan granules, a TCM, as the adjuvant treatment of community-acquired drug-resistant bacterial pneumonia (CDBP) caused by *Streptococcus pneumoniae*.

Lu et al. comprehensively explore the combination of pattern recognition chemometrics [i.e., similarity analysis (SA), cluster analysis (CA), and principal component analysis (PCA)] and chromatographic fingerprint analysis for the identification of quality markers (Q-marker) that contribute to the antioxidant activity of *Chrysanthemum morifolium* (CM) cv. (Juhua). The Q-marker is explored by correlating the concentrations of main constituents found in the HPLC chromatograms and the *in-vitro* radical scavenging capacity. The developed model is further used to evaluate the quality of 30 flower head samples of *C. morifolium*. The authors conclude that the combination of HPLC fingerprinting, *in-vitro* anti-oxidant activity evaluation, and chemometrics explain the therapeutic material basis and discovered Q-markers, providing a more comprehensive quality assessment of *C. morifolium*.

Rao et al. apply chromatographic fingerprinting based on UHPLC-Q-TOF MS and chemometrics of partial least square regression (PLSR), gray relational analysis models, and Spearman’s rank correlation coefficient (SRCC) to discover active compounds in herbal medicine, *Zanthoxylum nitidum* (Roxb.) DC. Using this approach, a total of 48 compounds are identified or tentatively characterized, which include 37 alkaloids, seven coumarins, three phenolic acids, two flavonoids, and one lignan. This study also predicts that some compounds namely nitidine, chelerythrine, hesperidin, and oxynitidine are potential anti-inflammatory compounds.

Another study applying a combination of UPLC-TOF-MS and chemometrics (i.e., pattern recognition and multivariate statistical analysis) is reported by Li et al. to discover the markers in TCM of Wutou decoction, used for the treatment of rheumatoid arthritis. Using Pearson correlation analysis, Q-markers of aconitine, l-ephedrine, l-methylephedrine, quercetin, albiflorin, paeoniflorigenone, astragaline A, astragaloside II, glycyrrhetic acid, glycyrrhizic acid, licurazide, and isoliquiritigenin are determined to be key pharmacological components that regulate the metabolism of rheumatoid arthritis in rats.

Liu et al. explore a metabolomics approach based on desorption electrospray ionization mass spectrometry imaging (DESI-MSI) and chemometrics of PCA and partial least square-discriminant analysis (PLS-DA) for the investigation of a series of Aconitum alkaloids and for exploring the potential metabolic markers intended to understand the differentiation between raw and processed Fuzi, a herbal medicine used for the treatment of various diseases. Forty-two metabolic markers are identified to discriminate raw and steam-processed Fuzi. Six alkaloids, namely mesaconitine, aconitine, hypaconitine, benzoylmesaconine, benzoylaconine, and benzoylhypaconine can be used as markers for the differentiation of raw and processed Fuzi. DESI-MSI, combined with metabolomics, provides an efficient method to visualize the changeable rules and screen metabolic markers of Aconitum alkaloids during processing.

Overall, three approaches (single component analysis, metabolic fingerprinting, and metabolomics studies) are used by researchers to discover chemical markers related to biological activities, identification, differentiation, and classification of HDs. Furthermore, to produce reproducible results, these approaches must be properly validated before their routine applications.

Finally, research on HD derived from natural sources must rely on careful design, thoughtful execution, and detailed reporting of the study focusing on the pharmacological or biological activities of active compounds extracted from HDs. Heinrich et al. provide a “Four Pillars of Best Practice” as a reference/guideline for articles submitted to scientific journals focusing on pharmacology and ethnobotany of HDs, including (1) pharmacological requirements (i.e., traditional context, testing of the therapeutically relevant dose range, and credible experimental models), (2) composition requirements of HD (chemical and botanical compositions), (3) basic experimental and ethical requirements, and (4) specific requirements in relation to article types (Heinrich et al., 2020).
